# What can animal studies tell us about EWB and AD

**DOI:** 10.1002/alz.086268

**Published:** 2025-01-03

**Authors:** Kuan Hong Wang

**Affiliations:** ^1^ University of Rochester Medical Center, Rochester, NY USA

## Abstract

Emotional well‐being (EWB) is considered to play an important role in the health of individuals over their lifespan. Clinical studies suggest an association between EWB and the risk or progression of AD. However, the mechanistic link and causal relationship between EWB and AD remain unknown, due to limited experimental access and control of the underlying brain processes in human. Animal models offer powerful tools for neural circuit analysis and control of AD genetic risk factors, but subjective feelings of EWB cannot be assessed through self‐report. To study EWB across species, we adopt a conceptual framework that views emotions as measurable states of brain networks that integrate exteroceptive and interoceptive affective stimuli and cause multiple cognitive, somatic and behavioral changes. Moreover, recent neuroanatomical and functional imaging studies have revealed evolutionarily conserved brain circuits, including mesocortical association areas and their connections with subcortical limbic areas, underlying the experienced and evaluative components of EWB. We will review recent progress in animal models to elucidate the functional activities of these circuits in emotional processing, the vulnerability of these circuits to AD pathology, and potential neuromodulation strategies to improve circuit functions. We will also offer our perspectives on the challenges and opportunities to link these neural representations to EWB and AD risk.

